# Cysteine Substitution and Calcium-Binding Mutations in *FBN1* cbEGF-Like Domains Are Associated With Severe Ocular Involvement in Patients With Congenital Ectopia Lentis

**DOI:** 10.3389/fcell.2021.816397

**Published:** 2022-02-14

**Authors:** Min Zhang, Zexu Chen, Tianhui Chen, Xiaodong Sun, Yongxiang Jiang

**Affiliations:** ^1^ Department of Ophthalmology and Vision Science, Eye and ENT Hospital of Fudan University, Shanghai, China; ^2^ NHC Key Laboratory of Myopia (Fudan University), Key Laboratory of Myopia, Chinese Academy of Medical Sciences, and Key Laboratory of Visual Impairment and Restoration of Shanghai, Shanghai, China; ^3^ Department of Ophthalmology, Shanghai General Hospital, Shanghai Jiao Tong University School of Medicine, Shanghai, China

**Keywords:** cbEGF-like, FBN1, cysteine, congenital ectopia lentis, marfan syndrome

## Abstract

**Purpose:** To investigate the clinical manifestations of congenital ectopia lentis (CEL) in patients with fibrillin (*FBN1*) calcium-binding epidermal growth factor (cbEGF)-like mutations.

**Design:** Retrospective cohort study.

**Methods:** Consecutive 68 CEL probands with *FBN1* cbEGF-like mutations were recruited, mostly comprising Marfan syndrome (MFS) patients. Patients were classified into the cysteine group (*n* = 43), calcium (Ca^2+^)-binding group (*n* = 13) or the others (*n* = 12) according to their genotypes. Ocular biometrics, morbidities and visual performance were compared among different mutation groups. Linear regression was used to evaluate the risk factors for axial length (AL) elongation.

**Results:** With age-adjustment, cysteine substitution and Ca^2+^-binding mutations positively contributed to AL elongation (standardized coefficient: 0.410 and 0.367, *p* = 0.008 and 0.017, respectively). In addition, cataract formation was more frequently detected in patients with Ca^2+^-binding mutations (observed *n* = 3, expected n = 1.0; *p* = 0.036). Patients with cysteine substitutions had the poorest preoperative visual acuity among the three groups (*p* = 0.012) and did not recover as well as other patients. More MFS diagnoses were made in patients with cysteine substitutions (observed *n* = 16, expected *n* = 12.6), while ectopia lentis syndrome was detected more often in patients with cbEGF-like mutations out of the functional regions (observed n = 6, expected n = 2.5; *p* = 0.023).

**Conclusion:** Compared with patients with cbEGF-like mutations out of functional regions, patients with cysteine substitutions or Ca^2+^-binding mutations had longer ALs with age adjustment, poorer ocular involvement, visual performance, and systematic manifestations.

## Introduction

Congenital ectopia lentis (CEL), or congenital lens subluxation, is a result of inheritable zonular dysplasia, and is the second leading cause of pediatric lens surgery after congenital cataracts. CEL can be an isolated ocular disease known as ectopia lentis syndrome (ELS) or be secondary to systematic disorders, such as Marfan syndrome (MFS), and both pathologies can be caused by fibrillin-1 (*FBN1*) mutations (Faivre et al., 2008).

**FIGURE 1:**
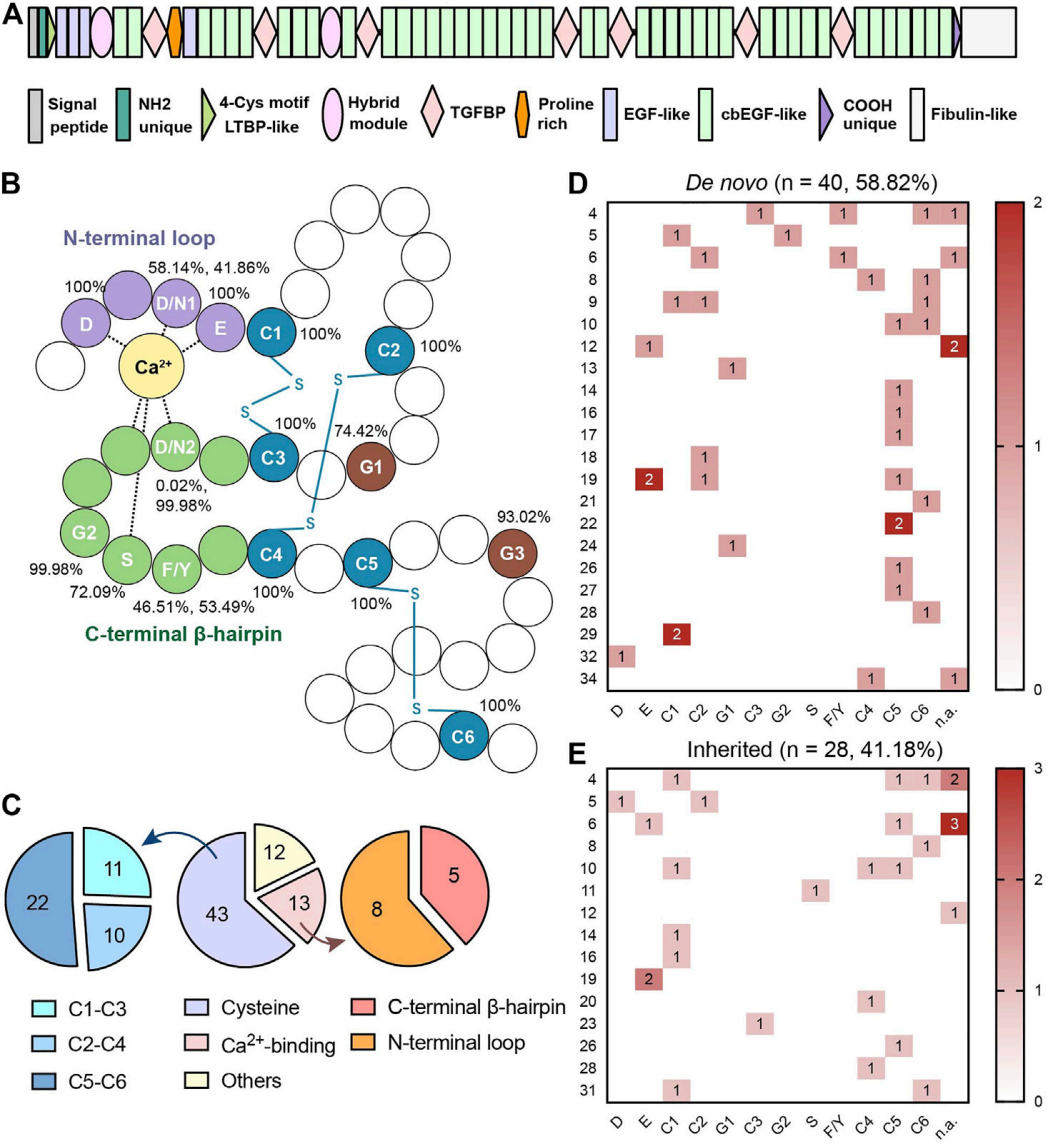
**(A)** Schematic diagram of FBN1 with forty-three cbEGF-like domains. **(B)** Schematic diagram of cbEGF-like domain. The N-terminal loop, C-terminal β-harpin and cysteine residues were remarked in purple, green and blue, respectively. All the conservative amino acid residues were annotated with abbreviations in white. Disulfide bonds (-S-S-) are annotated in blue. Percentages of conservative amino acid residues in different cbEGF-like domains in FBN1 peptides are also listed. The same conservative amino acids are numbered according to the sequence. **(C)** Distribution of mutations in different regions in cbEGF-like domains. Both cysteine pairs and Ca^2+^-binding regions were termed as functional regions. Cysteine substitutions and Ca^2+^-binding mutations were further divided into 3 pairs and 2 terminals, respectively. **(D,E)** are heatmaps of conservative amino acids mutations in patients with *de novo* mutations and with inherited mutations, respectively. Each line indicates one cbEGF-like domain in FBN1 and each column indicates one conservative amino acid. Each line indicates one cbEGF-like domain in FBN1 and each column indicates one conservative amino acid. cbEGF-like 11–18 fall into the neonatal region of FBN1. D = aspartic acid, N = asparagine, E = glutamic acid, C = cysteine, G = glycine, S = serine, F = phenylalanine, Y = tyrosine. The numbers following the amino acid indicated its order.

FBN1 is a cysteine-rich glycoprotein that serves as the principal structural component of microfibrils, contributing to the force-bearing capacity of zonules in the eyes and other connective tissue throughout the body. Calcium-binding epidermal growth factor (cbEGF)-like domains are the most common domains of FBN1 (Figure 1A). This consensus sequence is especially important for FBN1-to-microfibril assembly (Schrijver et al., 1999; Smallridge et al., 1999; Hilhorst-Hofstee et al., 2010; Schrenk et al., 2018). It relies on two special functional regions, the conserved cysteines and calcium-binding (Ca^2+^-binding) sequences, to construct a characteristic rigid rod-like shape (Haller et al., 2020). Disulfide bonds are formed among the six cysteine residues in a C1-C3, C2-C4, and C5-C6 pattern, which uniquely orchestrates protein folding. The Ca^2+^-binding sequence includes a N-terminal loop and a C-terminal β-hairpin. They contribute to mechanosensitive calcium-binding dynamics and the further stabilization of FBN1 (Schrenk et al., 2018). These two evolutionarily conserved functional regions extend alongside the cbEGF-like repeats for microfibril integrity and provide protection against proteolysis (Schrenk et al., 2018; Haller et al., 2020). Both of them are key functional regions in cbEGF-like domains (Figure 1B).

Around 60% of *FBN1* missense mutations occur in cbEGF-like domains (Haller et al., 2020; Chen et al., 2021a). These mutations are predicted to disrupt disulfide bonds or reduce calcium binding by removing cysteines or a side-chain ligand for calcium (McGettrick et al., 2000). Clinical manifestations, including cardiovascular (Wu et al., 2020), skeletal, and ophthalmic (Chen et al., 2021b) disorders, of patients with general *FBN1* mutations were widely analyzed. Cardiovascular problems have often been reported to be associated with cysteine substitutions in cbEGF-like domains (Kühne et al., 2013), but there is a lack of targeted research on the correlations between genotype and phenotype for cbEGF-like mutations and ocular lesions.

In addition, mutations in cbEGF-like domains 11–18, i.e., eight cbEGF-like domains in the neonatal region [exons 24–32, according to next-generation sequencing (NGS)], generally accounted for about 17% of missense mutations (Kühne et al., 2013; Haller et al., 2020; Wu et al., 2020). This region is named after its severity to frequently cause death before 2 years of age (Child, 2017). Severe cardiovascular involvement is reported to be associated with neonatal forms (Wu et al., 2020), especially those with cysteine substitution (Child, 2017). These cbEGF-like mutations in the neonatal region are systematic and are probably responsible for ocular microfibril disorganization and ophthalmic pathologies. Clinical observation is needed on this topic.

In this study, we reviewed the pathology of CEL patients with missense cbEGF-like mutations in *FBN1*. Most of them received surgical treatment and were followed up. We wished to address the following questions: 1) What are the ocular characteristics of CEL patients with cbEGF-like mutations? 2) Do patients with mutations in cysteine residues, Ca^2+^-binding sequences, and other sequences have different ocular biometrics and different distributions of ocular comorbidities, and do they have similar ocular involvement and systematic manifestation? 3) Do patients with cbEGF-like mutations in neonatal regions have ocular involvement earlier than patients with other cbEGF-like mutations?

## Methods

Consecutive CEL probands, mostly MFS patients, that visited our Department of Ophthalmology from March 2017 to March 2021 were initially recruited to this retrospective clinical observational case series (*N* = 170). The general inclusion and exclusion criteria were as described in published works (Chen et al., 2021a; Chen et al., 2021b). Briefly, CEL probands with a medical history of ocular trauma or surgeries were excluded.

The study was approved by the Human Research Ethics Committee of the Eye and ENT Hospital of Fudan University (no. 2020126–1) and performed with adherence to the tenets of the Declaration of Helsinki. It was also registered with the Chinese Clinical Trial Registry (ChiCTR2000039132). Written informed consent was obtained from all participants or their guardians before peripheral blood samples were collected.

### Ophthalmic Examination

Full ophthalmic examinations were performed on all CEL probands, and their medical histories were evaluated. The classification of lens subluxation directions is shown in Supplementary Figure S1A **.** The severity of lens subluxation was measured as described before (Chen et al., 2021c; Zhang et al., 2021) (Supplementary Figure S1B). Briefly, the curvature degree of the ring (pupil)-ring (lens) cross was measured with the pupil dilated to an 8-mm-diameter under slit-lamp examination. Curvature less than 180-, 180- to 270- and over 270-degrees were defined as mild, moderate and severe lens subluxation, respectively. Anterior segmental biometrics and axial length (AL) were measured using a partial coherence interferometry (iolmaster 700, Carl Zeiss Meditec AG, Jena, Germany) and a rotating Scheimpflug camera (Pentacam AXL, Oculus GmbH, Wetzlar, Germany). Ocular comorbidities were also detected by B-scan ultrasound and ultrasound biomicroscopy (MD-300L, 50-MHz probe transducer; Meda Co., Ltd., Tianjin, China).

### Genetic Screening

Peripheral blood samples underwent panel-based NGS (Amplicon Gene, Shanghai, China) for the exon sequences of 289 genes of common inherited anterior eye diseases (Supplementary Table S1). For patients with undetected pathogenic mutations but suspected *FBN1* mutations, multiplex ligation-dependent probe amplification (MLPA) of this gene was performed using SALSA MLPA Probemix Kits (P065-C1/P066-C1, MRC-Holland, Amsterdam, Netherlands).

The frequencies of identified variants were annotated through the Genome Aggregation Database (https://gnomad.broadinstitute.org/). Pathogenicity was also predicted by in silico predictive algorithms (SIFT, PolyPhen and Condel) using an integrated online software, the Ensembl Variant Effect Predictor (http://uswest.ensembl.org/info/docs/tools/vep/index.html).

### Genotype Classification

The causality nature of each *FBN1* mutation was evaluated using Ghent-2 criteria (Loeys et al., 2010). All mutations were classified following the American College of Medical Genetics and Genomics guidelines (Richards et al., 2015). All *FBN1* missense mutations were reviewed, and splicing variants and premature termination codons were excluded. Missense mutations were further classified based on the amino acid changes, location, and protein domains. The UMD-FBN1 database (http://umd.be/FBN1/) was referred to for the mapping of 43 cbEGF-like domains. Some special regions were annotated, such as the Ca^2+^-binding regions in the cbEGF-like domain (including N-terminal loop and C-terminal β-hairpin) (Smallridge et al., 1999; McGettrick et al., 2000; Haller et al., 2020) and the six conservative cysteine residues (C1-C6) (Suk et al., 2004).

### Patients Selection and Diagnoses

Probands with heterozygous pathogenic or likely pathogenic *FBN1* mutations were selected as shown in the flow chart **(** Supplementary Figure S2 **)**. Only the probands of pedigrees were recruited in this study. Considering high binocular correlation in these patients, as was shown in our previous study (Chen et al., 2021b), only one random eye from each proband were studied to avoid selection bias.

The systematic diagnosis of MFS was based on Ghent-2 nosology (Loeys et al., 2010). ELS was diagnosed in CEL adults with no history of cardiovascular disorders to avoid confounding. Otherwise, the patients were annotated as potential MFS cases.

### Surgical Treatment and Postoperative Follow-Ups

CEL patients underwent modified capsular tension ring (MCTR) and intraocular lens (IOL) in-the-bag implantation, as previously described (Chen et al., 2021c). Generally, the lens was aspired, but the capsular bag was preserved. The MCTR was sutured to the sclera through a sulcus by 9–0 polypropylene with the modified knotless Z-suture technique.

These patients were followed up in our out-patient department; surgical complications, such as retinal detachment, were reviewed, and visual records were obtained. The best corrected visual acuity (BCVA) was only recorded if posterior capsular opacification was not detected or was treated with Nd:YAG laser.

### Statistical Analyses

The distributions of ocular biometric parameters were tested for normality with the Shapiro-Wilk test. The Kruskal-Wallis test with Bonferroni correction was applied to compare the parameters among different mutation groups, and patients with and without neonatal mutations were compared with the Wilcoxon Mann-Whitney test. Spearman’s correlation test was used to explore the relationships among ocular biometrics and ages. The related-samples Wilcoxon signed-rank test was utilized to test BCVA changes before and after surgical treatment. BCVA records of patients with a medical history of retinal detachment were excluded in this analysis. Chi-square test or Fisher’s exact test was employed to compare the direction and severity of lens subluxation and the incidence of ocular comorbidities, as appropriate. Unmeasurable or unreliable values due to incoordination or incorporation were annotated as missing. Linear regression was used to identify the risk factors for AL elongation. *p* < 0.05 was considered statistically significant, and statistical analyses were performed using SPSS for Mac (version 26, 64-bit edition, IBM Corp, Armonk, NY, United States).

## Results

### Cohort Characteristics and Phenotypic Summaries

A total of 68 (44.12%) probands were included in the genotype-phenotype analyses of the cbEGF-like mutations. The mean age of our cohort of 39 boys/men and 29 girls/women was 12.47 ± 12.63 (median: 7, range: 2–58) years old. Table 1 presents the demographics and clinical characteristics of the enrolled eyes. Among these probands, 28 (41.18%) patients had cbEGF-like mutations inherited from their parents, while 40 (58.82%) had *de novo* mutations. Our spontaneous mutation rate was much higher than that previously reported (Madar et al., 2019).

**TABLE 1:** Demographics and clinical characteristics of patients with enrolled eyes.

Characteristics				Counts (%) or mean ± SD (median, range)	
Demographics	Male/female			39 (57.35)/29 (42.65)	
	Age (years old)	≤20		12.47 ± 12.63 (2 to 58, 7)	53 (77.94)
		>20			15 (22.06)
	Neonatal/others			13 (19.12)/55 (80.88)	
Ocular phenotypes	Bilateral/unilateral subluxation			67 (98.53)/1 (1.47)	
	Right/left			32 (47.06)/36 (52.94)	
	Comorbidity	MSP		8 (11.76)	
		Cataract		5 (7.35)	
		Strabismus		6 (8.82)	
		Staphyloma		20 (29.41)	
		Glaucoma		3 (4.41)	
		Ciliary body cyst		4 (5.88)	
		Megalocornea		4 (5.88)	
	AL (mm), n = 64)			25.06 ± 3.03 (24.27, 21.11 to 33.62)	
	ACD (mm), n = 43			3.21 ± 0.45 (3.24, 1.80 to 4.46)	
	LT (mm), n = 30			3.83 ± 0.98 (3.83, 1.36 to 5.90)	
	WTW (mm), n = 37			12.21 ± 0.56 (12.20, 11.2 to 13.5)	
	Corneal biometrics	Anterior K1 (D), n = 52		39.71 ± 1.64 (39.85, 36.3 to 43.7)	
		Anterior K2 (D), n = 52		41.45 ± 1.74 (41.40, 38.2 to 46.5)	
		Anterior Km (D), n = 59		40.60 ± 1.72 (40.60, 37.7 to 46.0)	
		Total K1 (D), n = 50		39.34 ± 1.88 (39.50, 35.9 to 45.4)	
		Total K2 (D), n = 50		41.22 ± 1.99 (40.90, 37.6 to 46.5)	
		Total Km (D), n = 56		40.26 ± 1.79 (40.10, 36.9 to 45.9)	
		TCRP (D), n = 55		39.89 ± 1.59 (40.10, 36.7 to 45.1)	
		SA (μm), n = 56		0.096 ± 0.133 (0.091, -0.415 to 0.584)	
		HOA (μm), n = 56		0.233 ± 0.281 (0.171, 0.082 to 1.650)	
		CCT (μm), n = 59		542.86 ± 41.25 (541, 458 to 640)	
		Endothelial cell count (/mm^2^), n = 59		3273.05 ± 437.61 (3307, 2013 to 4365)	
Surgical treatment	Treatment/naïve			66 (97.06)/2 (2.94)	
	Postoperative RD^a^			3 (4.55)	
	Follow-up of visual outcomes^b^, n = 47	Duration (months)		13.91 ± 9.38, 12 (1 to 24)	
		BCVA (LogMAR)	Baseline	0.71 ± 0.48, 0.52 (0.10 to 2.00)	
			Postoperative	0.19 ± 0.22, 0.15 (0.00 to 1.30)	
			*P* value^c^	<0.001	

^^^Others refer to lens tremor or anterior or posterior subluxation.

SA, spherical aberration; HOA, high-order aberrations; MSP, microspherophakia; AL, axial length; ACD, anterior chamber depth; LT, lens thickness; WTW, white-to-white, K1 = minimal corneal power, K2 = maximal corneal power, Km = mean corneal power, TCRP, total corneal refractive power (centered at corneal apex), BCVA, best corrected visual acuity; RD, retinal detachment.

a With or without postoperative RD, complications were recorded in all 66 patients.

b Complete visual outcomes were obtained in 49 patients. Records of 2 patients with medical history of retinal detachment were excluded and visual outcomes of 47 patients were presented here.

c *p* value was reported by related-samples Wilcoxon signed-rank test.

Nearly all the patients had bilateral lens subluxation (*n* = 67, 98.53%) rather than unilateral lens subluxation. Almost half of the patients had lenses subluxated into the superior-nasal quadrant (*n* = 32, 47.06%) or nasal side (*n* = 9, 13.24%). Most patients had lens subluxation within three quadrants (≤270°), including 24 (35.29%) mild cases and 34 (50.00%) moderate cases. The most common ocular comorbidity was posterior staphyloma (*n* = 20, 29.41%). Table 1 also presents the ocular manifestations of all enrolled patients, including long AL (25.06 *±* 3.03 mm), flat cornea (total corneal Km = 40.26 ± 1.79 D), and reduced corneal spherical aberration (SA; 0.096 ± 0.133 μm).

There were 66 patients who received surgical treatment (Table 1). Of the remaining two treatment-naïve patients, one had a case of lens subluxation that was not severe enough to warrant intervention, and the other was poorly cooperative. During the maximum 24-months follow-up period, three cases (4.55%) were complicated with postoperative retinal detachment. In patients with no severe postoperative complications, the median LogMAR BCVA recovered from 0.52 to 0.15 (*p* < 0.001).

The genotypes, phenotypes and segregation of all enrolled patients are summarized in Supplementary Tables S2–4.

### Distribution of Mutation Sites in cbEGF-like Domains

Out of the 47 EGF modules in *FBN1*, 43 contained the cbEGF consensus sequence (Schrenk et al., 2018). This study showed that all missense mutations were distributed over 27 cbEGF-like domains (27/43 = 62.79%). Fifty-six (82.35%) patients had mutations in functional regions **(** Figure 1C **)**, and a comparative percentage of conservative amino acid mutations was also detected in patients with *de novo* mutations (35/40 = 87.50%; Figure 1D). There were 43 patients (43/68 = 63.24%) with cysteine substitutions in cbEGF-like domains, and nearly half of them (22/43, 51.16%) had mutations in the C5-C6 pair. The C5-C6 pair was also the top *de novo* and the top inherited mutation hotspot (15/40 = 37.5%, Figures 1D,E). Specifically, the most prevalent mutation was c.4096G > A/p.E1366K in cbEGF 19 (4/68 = 5.88%).

### Ocular Biometrics and Age-Related Changes With cbEGF-like Mutations

To better evaluate the ocular biometrics of patients with different cbEGF-like mutations and determine the potential role of the *FBN1* genotypes, the age-related effects on ocular biometrics first had to be determined and removed.

Five ocular biometrics were found to be significantly correlated with age: corneal endothelial cell counts (r_s_ = −0.489, *p* < 0.001, *n* = 59), AL (r_s_ = 0.407, *p* = 0.001, *n* = 64), total corneal K1 (r_s_ = 0.294, *p* = 0.038, *n* = 50), SA (r_s_ = 0.364, *p* = 0.006, *n* = 56), and higher-order aberrations (r_s_ = −0.277, *p* = 0.039, *n* = 56, Supplementary Figure S3). Furthermore, age-adjusted linear regression showed that cysteine substitution (*p* = 0.008) and Ca^2+^-binding mutations (*p* = 0.017) were significantly and positively associated with AL elongation, even if no differences in age or AL distribution were detected among the three mutation groups (Table 2).

**TABLE 2:** Mutations in Cysteine or Calcium-binding related regions contributed to AL-elongation.

Mean ± SD (median, range) or observed counts (expected counts)		Functional regions (*n* = 53)		Others (*n* = 11)	*p* values
		Cysteine (*n* = 41)	Ca^2+^-binding (*n* = 12)		
Age (years old)		11.60 ± 9.81 (8, 2–41)	12.77 ± 15.92 (6, 3–58)	15.25 ± 17.78 (6.5, 3–54)	0.771^a^
AL (mm)	≤26 (*n* = 43)	26 (27.6)	6 (8.1)	11 (7.3)	0.091^b^
	26–28 (*n* = 6)	4 (3.8)	2 (1.1)	0 (1.0)	
	>28 (*n* = 15)	11 (9.6)	4 (2.8)	0 (2.6)	
**Linear regression: AL (mm)**	**Standardized coefficient**		***t***		***p* values^c^**
Constant	—		23.146		<0.001
Age (years old)	0.354		3.066		0.003
Cysteine	0.410		2.721		0.008
Ca^2+^-binding	0.367		2.453		0.017

AL, axial length. There were 4 AL values missing, *n* = 64.

a *p* value was reported by Kruskal-Wallis test.

b *p* value was reported by Fisher exact test.

c *p* values were reported by linear regression and the dependent variable was AL (mm).

The only ocular parameter found to be significantly different among the mutation groups was SA **(** *p* = 0.009, Supplementary Figure S4A). Patients with cysteine substitutions, in particular, had a lower SA than those with cbEGF-like mutations out of the functional regions (*p* = 0.007). Though there was no significant age difference among the three mutation groups (*p* = 0.771, Supplementary Figure S4B), linear regression showed that for SA reduction, age was a significant factor (*p* < 0.001) but not cysteine substitution (*p* = 0.174). The reduced SA in the cysteine substitution group could be explained by lower age of patients with cysteine substitutions rather than effects of cysteine substitutions themselves.

### Lens Subluxation and Ocular Comorbidities With cbEGF-like Mutations

**FIGURE 2:**
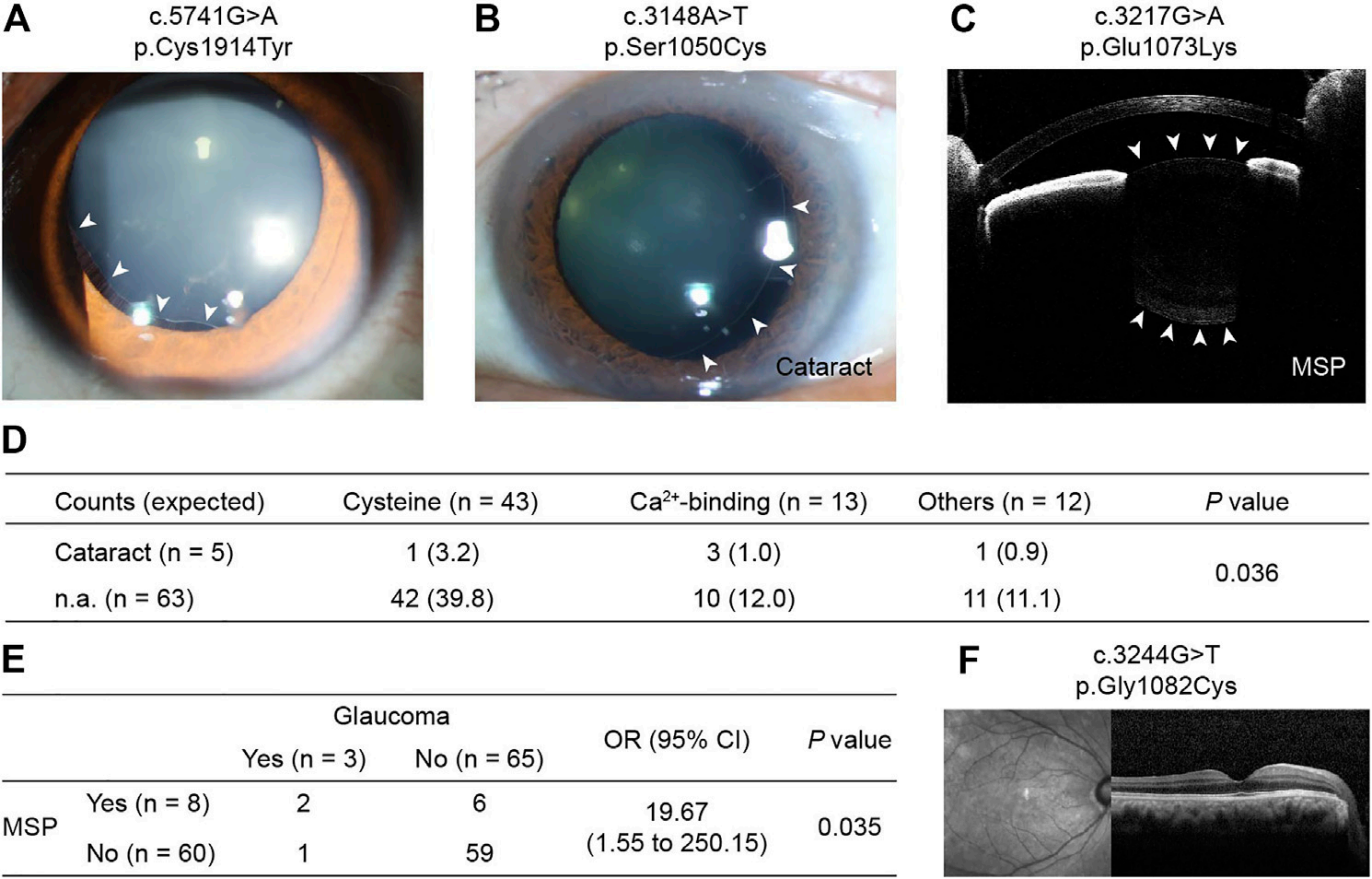
Photos of CEL in patients with *FBN1* cbEGF-like mutations and their ocular comorbidities. **(A)** Right eye of patient no. 7 with mild lens subluxation into the superior-temporal quadrant. **(B)** Left eye of patient no. 20 with moderate lens subluxation and cataract. **(C)** Right eye of patient no. 47 with severe lens subluxation and MSP. White arrowhead in **(A,C)** indicate the border of lens. **(D)** Inclination of cataract formation was detected in patients with Ca^2+^-binding mutations. *p* values were reported by Fisher’s exact test. **(E)** Consistency of MSP and glaucoma in CEL patients. MSP = microspherophakia. OR = odds ratio, CI = confidence interval. *p* value was reported by Fisher’s exact test. **(F)** Optical coherence tomography of patient no. 2 with normal macular structure. MSP = microspherophakia.

Figures 2A–C provides examples of lens subluxation with and without ocular comorbidities. No significant differences in subluxation direction (*p* = 0.590) or subluxation severity (*p* = 0.911) were detected among the three mutation groups (Supplementary Table S5).

Among CEL patients with ocular comorbidities, one 6-year-old boy with a cataract was noted (c.3921T > G/p.Cys1307Trp). Advanced analysis showed that there was an increase in cataract formation in patients with Ca^2+^-binding mutations (*p* = 0.036, Figure 2D). However, the three cataract patients with Ca^2+^-binding mutations were 22, 31, and 58 years old, and they all had quite long AL (28.73, 29.61, and 30.09 mm, respectively). Therefore, whether cataract formation can be directly attributed to mutations or secondarily to the patients’ relatively advanced age or high myopia is uncertain. Although no inclination of other ocular comorbidities was detected in patients with functional region mutations (Supplementary Table S5), MSP and glaucoma were often observed consistently **(** Figure 2E **)**, indicating the high susceptibility of MSP cases to glaucoma.

Incidentally, no retinal abnormalities were detected in any patient on optical coherence tomography (OCT) (Figure 2F), except for postoperative retinal detachment in three cases.

### Visual Performance and Systematic Diagnoses of Patients With cbEGF-like Mutations

**FIGURE 3:**
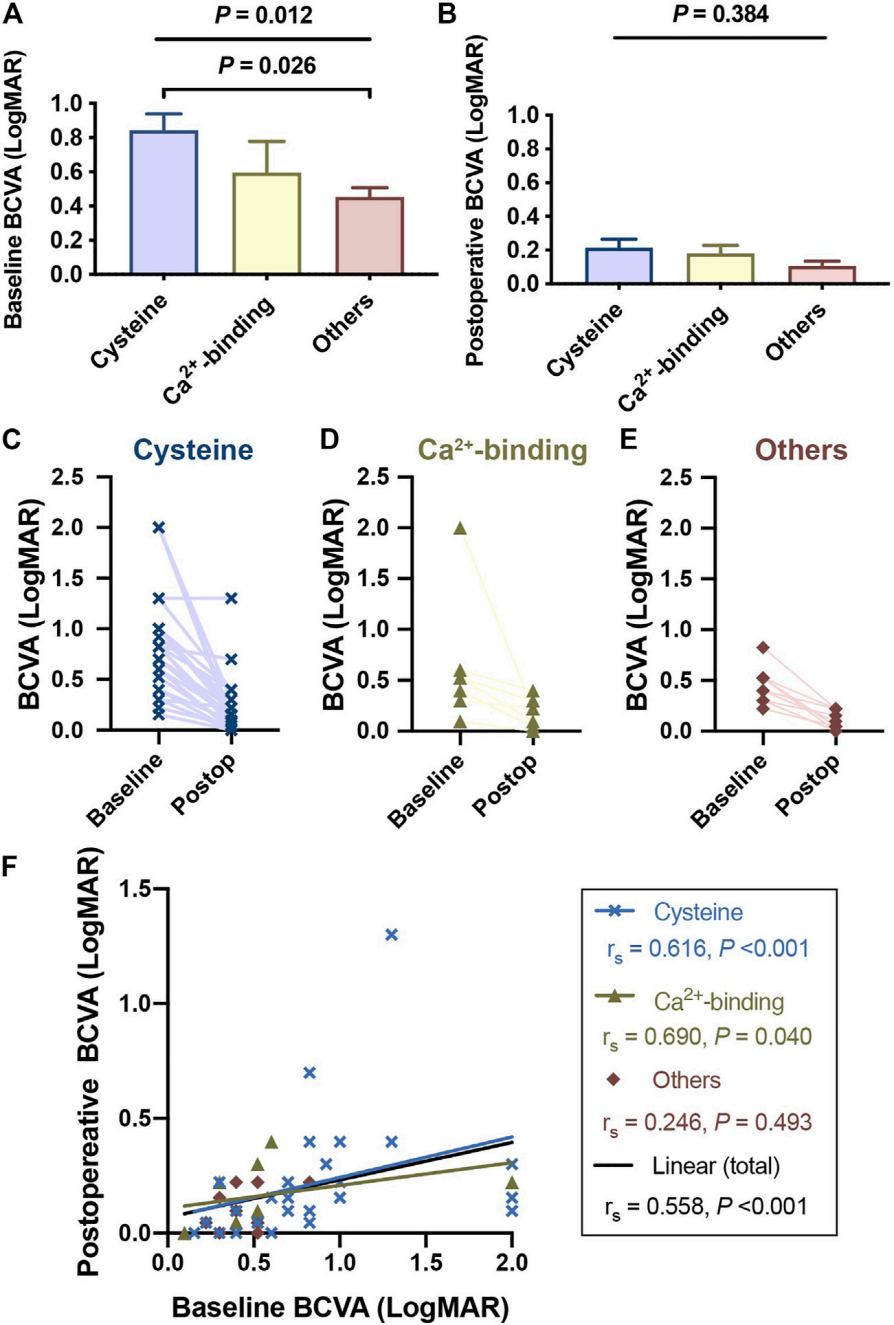
Patients with cysteine substitutions had the poorest visual performance among different functional region groups. Complete visual outcomes before and after surgical treatments were obtained in 49 patients. Records of 2 patients with the medical history of retinal detachment were excluded and visual outcomes of 47 patients were presented here. **(A)** Patients with cysteine substitutions had the poorest visual performance among different functional region groups, reported by Kruskal-Wallis test (with Bonferroni correction) at the baseline. **(B)** Though this difference was not significant anymore after the surgical treatment, patients who had cbEGF-like mutations out of functional regions still tended to have better visual outcomes. **(C,E)** showed the BCVA changes of each patient with different cbEGF-like mutations before and after the surgical treatment. Postop = postoperative. **(F)** Linear correlation between the baseline BCVA and postoperative BCVA. The blue line and the black line indicated the linear correlations of these two variables in the patients with cysteine substitution and in all study populations, respectively. r_s_ and *p* values were reported by Spearman’s correlation test.

Patients with cysteine substitutions had the poorest BCVA among the different mutation groups (*p* = 0.012 at baseline, Figure 3A; *p* = 0.384 postoperatively, Figure 3B), and there was obvious visual improvement before and after surgical treatment (*p* < 0.001). The median LogMAR BCVA recovered from 0.76 to 0.15 (*p* < 0.001) in the cysteine group, from 0.52 to 0.22 in the Ca^2+^-binding group (*p* = 0.008), and from 0.46 to 0.07 in the remaining patients (*p* = 0.005). Patients in the different groups were followed up over similar durations (*p* = 0.926, Supplementary Table S6). Although the postoperative BCVA was not significantly different among the different mutation groups (*p* = 0.330), the values showed a decreasing trend in the order of cysteine group, Ca^2+^-binding group, and others.

Similar results were detected in the systematic diagnoses. More MFS diagnoses were found in the cysteine group (observed *n* = 16, expected *n* = 12.6), while ELS was more often detected in patients with cbEGF-like mutations out of the functional regions (observed *n* = 6, expected n = 2.5; *p* = 0.023, Table 3). Patients with Ca^2+^-binding mutations tended to have similar diagnosis distributions to the overall study populations. These results indicate that patients with cysteine substitutions have the worst overall systematic manifestations in all patients with cbEGF-like mutations.

**TABLE 3:** Patients with cbEGF-like mutations in functional regions were more vulnerable to systematic disorders.

Observed counts (expected counts)		Diagnoses (*N* = 68)			*p* value
		potential MFS	MFS	ELS	
		(*n* = 34)	(*n* = 20)	(*n* = 14)	
Functional regions (*n* = 56)	Cysteine (*n* = 43)	23 (21.5)	16 (12.6)	4 (8.9)	0.023
	Ca^2+^-binding (*n* = 13)	7 (6.5)	2 (3.8)	4 (2.7)	
Others (*n* = 12)		4 (6.0)	2 (3.5)	6 (2.5)	

p value was reported by Fisher’s exact test. MFS, Marfan syndrome; ELS, Ectopia lens syndrome. Others refer to cbEGF-like mutations out of cbEGF-like functional regions.

### cbEGF-like Mutations in the Neonatal Region

**FIGURE 4:**
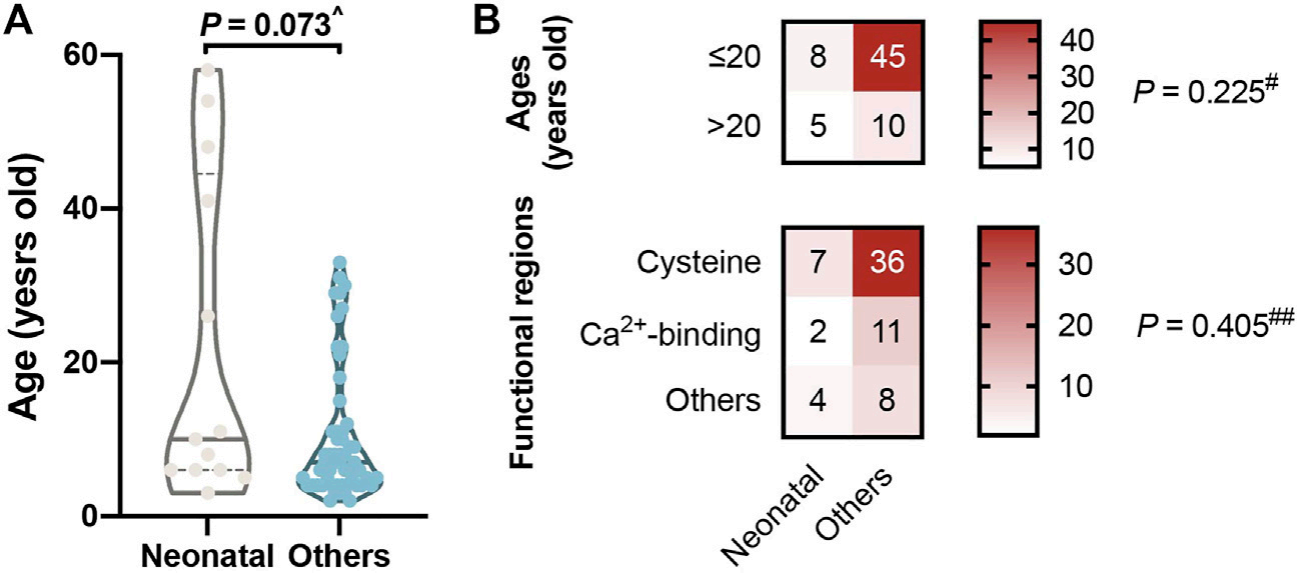
Mutations in neonatal regions (cbEGF like 11–18). **(A)** No significant difference in ages between the neonatal group and the others. It was surprising to find that our four oldest patients all had mutations in the neonatal group. **(B)** Neonatal mutations are distributed adequately among different age strata and functional regions. ^^^ *p* value reported by Wilcoxon Mann-Whitney test, ^#^ *p* value reported by Pearson Chi-square test with continuity correction and ^##^ *p* value reported by Fisher’s exact test.

A total of 13 (19.12%) patients had mutations that fell within the neonatal region (Table 1), comparable to the reported 17% percent of neonatal missense mutations in all *FBN1* missense mutations (Kühne et al., 2013). We were surprised to find that patients in the neonatal group were generally older than the others, although not significantly (*p* = 0.073; Figure 4A). One patient with lens subluxation was even diagnosed at 58 years of age (c.3148A > T, p. Ser1050Cys); he had lens subluxation combined with cataract but was only diagnosed as ELS. In addition, neonatal mutations are distributed adequately among different age strata and functional regions (*p* = 0.225 and 405, respectively; Figure 4B).

Detailed differences were further detected in cbEGF-like mutations in the neonatal region. A hotspot of cbEGF-like mutations in the neonatal region was cbEGF-like 12 (*n* = 4), but no patient’s carried cysteine substitutions. Instead, cysteines eliminations were found in all cbEGF-like 14 mutations (*n* = 3), which was the second hotspot in the neonatal region. Considering the other domains’ long-range cooperative dependence on cbEGF-like 12^19^, the importance of cysteines was again suggested. Furthermore, there were no mutations in cbEGF-like 13 detected in our patients, which may have contributed to its highest Ca^2+^-binding affinity and high-malignancy of the corresponding mutations.

## Discussion

In this study, we reviewed the ocular biometrics and medical histories of 68 CEL patients with cbEGF-like mutations in *FBN1*. The involvement of cysteine substitutions or Ca^2+^-binding mutations, along with the patients’ age, contributed to AL elongation. More severe ocular involvement and systematic manifestations were also found in these patients with mutations in functional regions. We showed that cysteine substitutions in cbEGF-like domains, along with Ca^2+^-binding mutations, were of great clinical significance in CEL patients.

We observed long AL (25.03 ± 3.04 mm), flat cornea (Km = 40.58 ± 1.71 D), reduced corneal SA (0.096 ± 0.133 μm), and frequent lens subluxation into the superior-nasal quadrant (*n* = 31, 45.59%) in CEL patients. These were consistent with our previous findings (Chen et al., 2018a; Chen et al., 2018b; Chen et al., 2021a; Chen et al., 2021b; Chen et al., 2021d) and reports of other MFS patients (Sandvik et al., 2019; Vanhonsebrouck et al., 2021). AL was found to be reasonably increased with aging (r_s_ = 0.407, *p* = 0.001, Supplementary Figure S3). The positive correlations between the corneal SA and ages in our patients (r_s_ = 0.364, *p* = 0.006) were also consistent with a study of a normal population (Navarro et al., 2013). However, we found that in patients with cbEGF-like mutations, the total corneal K1 increased with aging (r_s_ = 0.294, *p* = 0.038). This differs from the overall findings for MFS patients with lens subluxation (Chen et al., 2018a) or healthy children (He et al., 2021; Liu et al., 2021), indicating the specific role of cbEGF-like mutations, rather than general *FBN1* mutations, in age-related corneal changes.

Therefore, statistical comparisons of ocular biometrics among the different mutation groups should first be weighted for age. After age-adjustment, linear regression showed that both cysteine substitutions and Ca^2+^-binding mutations contributed to AL elongation **(** Table 2). The cysteine substitution even contributed to AL elongation more than Ca^2+^-binding mutations (standardized coefficient: 0.410 vs. 0.367). We also found that no patients with mutations out of the functional regions had ALs longer than 26 mm (Table 2). Hence, the findings re-confirmed the involvement of functional region mutations in ocular development.

In addition to the ocular biometrics, functional mutations in cbEGF-like domains also contributed to ocular comorbidities, such as cataracts, MSP, and glaucoma (Figure 2). In this study, patients with Ca^2+^-binding mutations, but not cysteine substitutions, tended to develop cataracts (*p* = 0.036, Figure 2D). The comorbidities of MSP and glaucoma were also detected. Glaucoma is considered to be secondary to MSP (Yu et al., 2020) and to lens subluxation (Zhou et al., 2021). We detected a significantly increased incidence of glaucoma development with MSP [odds ratio (OR): 19.67, *p* = 0.035; Figure 2E]. However, the incidence of glaucoma in MSP patients was reported to be 44–51% (Yu et al., 2020), much higher than those detected in our cohort (2/8 = 25%). This might be due to the posterior dislocation of MSP in these patients, as vitreous liquefaction at the base was often observed in those patients (Remulla and Tolentino, 2001). Interestingly, only one CEL patient with glaucoma but without MSP, had cysteine substitutions (c.2810G > A/p.Cys937Tyr). Once again, the functional region mutations caught our attention. More work is needed to clarify the underlying mechanisms of cbEGF-like mutations in zonular weakness and the lens subluxation-MSP-glaucoma axis.

We also found that patients with cysteine substitutions had poor visual performance. Previous 10-years reinvestigation reported that the visual potential of MFS patients was relatively good (Sandvik et al., 2019). Our results are consistent with this interpretation, as 41 (87.22%) patients had postoperative LogMAR BCVA of better than 0.3 (Figure 3). However, those with cysteine substitutions had the poorest preoperative LogMAR BCVA among the three groups and did not recover as well as those in the other two mutation groups (Figures 3C–F). This was difficult to explain, as no retinal abnormalities were detected in any patient given OCT (Figure 2F), except for three with postoperative retinal detachment (not included in the statistical analyses of visual performance). The fundus blood flow density of patients with cysteine substitutions might be affected, leading to defects in visual function. Amblyopia might also contribute to this situation. Advanced visual analyses of these patients’ data are needed to clarify the underlying mechanism.

Long-term follow-up also showed that the risk of developing vision-threatening complications, such as retinal detachment in MFS patients, was still much higher than in the normal population (Sandvik et al., 2019). In this study, three retinal detachment cases were detected (Sandvik et al., 2019). Though all three retinal detachment cases had cysteine substitutions, the surgical complications were rarely seen in general study populations, and it was difficult for us to investigate the correlation between functional region mutations and retinal detachment. All these patients had posterior staphyloma and had almost the longest ALs in our cohort. Thus, pathological myopia may take the responsibility for myopic rhegmatogenous retinal detachment (Ruiz-Medrano et al., 2019).

When we focused on cbEGF-like mutations in the neonatal regions, they were all found to occur in patients older than 2 years. This came from the recruitment methods of our study, as neonatal MFS with severe cardiovascular and skeletal abnormalities tend to be treated in the Department of Pediatrics rather than the Department of Ophthalmology. But it was surprising to find that our four oldest patients all had mutations in the neonatal group **(** Figure 4 **)**. The involvement of different functional region mutations and changes in cbEGF-like domain rigidity might contribute to this phenomenon. For example, the cbEGF-like 12/13 pair is located within the longest contiguous section of cbEGF-like domains, and a number of mutations in this pair are associated with the most severe neonatal MFS (Whiteman et al., 2007). In addition, cbEGF-like 13 possesses the highest Ca^2+^ affinity in any cbEGF-like investigated from FBN1 (Smallridge et al., 1999). Because cbEGF-Ca^2+^ affinity can be modulated by the domain that is linked to its N-terminus, the affinities of the cbEGF-like 13/14 pair are also substantially higher than those of the C-terminal region of FBN1 (Smallridge et al., 1999). Our observation was in accordance with these biomechanical characteristics, as no mutation was detected in cbEGF-like 13. Additionally, mutations other than cysteine substitutions in cbEGF-like 12 and cysteine substitutions in cbEGF-like 14 were the most prevalent in the neonatal regions. Combined with the report that cardiovascular disorders correlated with cysteine substitutions (Kühne et al., 2013), we propose that mutations of cbEGF-like 13 and cysteine substitutions in cbEGF-like 12, followed by cysteine substitutions in cbEGF-like 14, were the top three hazardous neonatal mutations.

There were some limitations in our study. 1) The postoperative visual outcomes of some patients were not rigorously or regularly recorded. This was due, to some extent, to the retrospective nature and dramatically increased postoperative BCVA. There were 22 patients (46.81%) who had near-term postoperative LogMAR BCAV <0.10 (fraction >32/40), some of whom were satisfied with the surgical outcomes and were lost in long-term follow-ups. 2) We still had some patients diagnosed with potential MFS, and the final confirmation was not achieved. This came from the fact that some of our patients were too young to fully exclude the cardiovascular disorders. The diagnoses of these patients can only be determined until adulthood. 3) Although Ca^2+^-binding regions and cysteine residues work together in maintaining cbEGF-like domain rigidity, they play different roles in binding calcium ions and forming disulfide bonds. It is reasonable for us to consider both of them as functional regions and compare the clinical manifestation of the patients between these two groups.

Overall, CEL patients with *FBN1* cbEGF-like mutations also had ocular characteristics of long AL, flat cornea, and reduced corneal SA. Compared with patients with cbEGF-like mutations out of functional regions, patients with cysteine substitutions and Ca^2+^-binding mutations had longer AL after age adjustment. They also had poorer ocular involvement, visual performance, and systematic manifestations. In our series, those with cbEGF-like mutations in the neonatal regions did not show earlier ocular involvement compared with patients with cbEGF-like mutations out of the neonatal regions.

## Data Availability

The raw data supporting the conclusion of this article will be made available by the authors, without undue reservation.
