# Supplementary material for: Cysteine Substitution and Calcium-Binding Mutations in FBN1 cbEGF-Like Domains Are Associated With Severe Ocular Involvement in Patients With Congenital Ectopia Lentis
Source: Front Cell Dev Biol. 2022 Feb 14;9:816397. doi: 10.3389/fcell.2021.816397 (PMC8882981; doi:10.3389/fcell.2021.816397)
Supplement: Supplementary file 1 [file Table1.docx]

**Supplementary Table S1.** 289 genes NGS Panel.

| AASS | ABCA1 | ABCB6 | ABHD12 | ACO2 | ADAMTS10 | ADAMTS17 | ADAMTS18 |
| --- | --- | --- | --- | --- | --- | --- | --- |
| ADAMTSL2 | ADAMTSL4 | AFG3L2 | AGBL1 | AGK | AGPS | AIRE | ALDH1A3 |
| ANTXR1 | APOA1 | ASB10 | ASPH | ATOH7 | ATXN1 | AUH | B3GLCT |
| B4GAT1 | BCOR | BEST1 | BFSP1 | BFSP2 | BMP4 | BMP7 | BTD |
| C12orf57 | C12orf65 | CASK | CAV1 | CAV2 | CBS | CDKN2B | CDKN2B-AS1 |
| CETP | CHD7 | CHMP4B | CHRDL1 | CHST6 | CISD2 | CLPB | CLU |
| COL11A1 | COL11A2 | COL15A1 | COL17A1 | COL18A1 | COL1A2 | COL2A1 | COL4A1 |
| COL4A3 | COL4A4 | COL4A5 | COL8A2 | COL9A1 | COL9A2 | COX7B | CPAMD8 |
| CRYAA | CRYAB | CRYBA1 | CRYBA2 | CRYBA4 | CRYBB1 | CRYBB2 | CRYBB3 |
| CRYGA | CRYGB | CRYGC | CRYGD | CRYGS | CTDP1 | CYP1B1 | CYP4V2 |
| DCN | DHCR7 | DNAJC19 | EDN3 | EDNRB | EGR1 | ELP4 | EPG5 |
| EPHA2 | EPYC | ERCC2 | ERCC6 | ESR1 | EYA1 | FAM126A | FBN1 |
| FBN2 | FGFR2 | FLNA | FOXC1 | FOXC2 | FOXD3 | FOXE3 | FOXF2 |
| FTL | FYCO1 | GALK1 | GALNS | GALT | GAS7 | GBA2 | GCNT2 |
| GDF3 | GDF6 | GEMIN4 | GFER | GJA1 | GJA3 | GJA8 | GLB1 |
| GLIS3 | GNAQ | GNPAT | GNPTG | GSN | GSTM1 | GSTT1 | GUSB |
| HCCS | HGSNAT | HMGB3 | HMX1 | HSF4 | IARS2 | IDUA | IKBKG |
| ITM2B | JAG1 | KCNJ13 | KERA | KRT12 | KRT3 | LAMB2 | LCAT |
| LDLR | LEMD2 | LGR4 | LIFR | LIM2 | LMX1B | LOXHD1 | LOXL1 |
| LSS | LTBP2 | LUM | MAB21L2 | MAF | MCOLN1 | MFN2 | MFRP |
| MIP | MIR184 | MIR204 | MMP2 | MSMO1 | MYLK | MYOC | NAA10 |
| NDP | NDUFB11 | NDUFS1 | NEUROG1 | NHS | NOD2 | NOTCH2 | NR2F1 |
| NTF4 | OCRL | OLFM2 | OPA1 | OPA3 | OPTN | OTX2 | OVOL2 |
| P3H2 | PAX2 | PAX3 | PAX6 | PEX1 | PEX10 | PEX11B | PEX12 |
| PEX13 | PEX14 | PEX16 | PEX19 | PEX2 | PEX26 | PEX3 | PEX5 |
| PEX6 | PEX7 | PIGN | PIKFYVE | PITX2 | PITX3 | PNPT1 | POLG |
| PRDM5 | PRPS1 | PRSS56 | PTCH1 | PXDN | RAB18 | RAB3GAP1 | RAB3GAP2 |
| RARB | RAX | RBP4 | RDH11 | RECQL4 | RET | RTN4IP1 | SALL1 |
| SALL2 | SBF2 | SEMA3E | SH3PXD2B | SHH | SIL1 | SIPA1L3 | SIX1 |
| SIX6 | SLC16A12 | SLC25A46 | SLC33A1 | SLC38A8 | SLC39A13 | SLC4A11 | SLC4A4 |
| SMAD3 | SMAD7 | SMOC1 | SNAI2 | SORD | SOX10 | SOX2 | SOX9 |
| SPG7 | SRD5A3 | STRA6 | STS | SUOX | TACSTD2 | TBC1D20 | TBK1 |
| TBX1 | TCF4 | TDRD7 | TEK | TENM3 | TFAP2A | TGFBI | TMCO1 |
| TMEM114 | TMEM126A | TMEM5 | TMEM70 | TMEM98 | TMX3 | TNFAIP3 | TRIM44 |
| TRPM3 | TSPAN12 | UBIAD1 | UNC45B | VAX1 | VCAN | VIM | VSX1 |
| VSX2 | WDR36 | WFS1 | WRN | YAP1 | ZEB1 | ZEB2 | ZFHX4 |
| ZNF469 |  |  |  |  |  |  |  |

NGS = next generation sequence.

**Supplementary Table S2.** Genotyping and ocular comorbidities of all enrolled patients.

| No. | Gender | Age (years old) | Eye | Family history | Gene | Variant | | Subluxation direction^^^ | Subluxation severity | Cataract | Strabismus | Staphyloma | Glaucoma | MSP | Ciliary body cyst | Megalocornea |
| --- | --- | --- | --- | --- | --- | --- | --- | --- | --- | --- | --- | --- | --- | --- | --- | --- |
|  |  |  |  |  |  | cDNA | Amino acid |  |  |  |  |  |  |  |  |  |
| 1 | F | 5 | OD | De novo | *FBN1* | c.1904A>G | p.Y635C | S-N | Moderate | N | N | N | N | N | N | N |
| 2 | F | 48 | OD | Inherited | *FBN1* | c.3244G>T | p.G1082C | O | Severe | N | N | N | Y | Y | N | N |
| 3 | F | 10 | OD | De novo | *FBN1* | c.1633C>T | p.R545C | N | Moderate | N | N | N | N | N | N | N |
| 4 | M | 3 | OS | De novo | *FBN1* | c.5504G>A | p.C1835Y | S-N | Mild | N | N | Y | N | N | N | N |
| 5 | M | 26 | OD | De novo | *FBN1* | c.3380G>T | p.G1127V | S-N | Mild | N | N | N | N | N | N | N |
| 6 | F | 6 | OS | De novo | *FBN1* | c.1638C>G | p.C546W | S-N | Mild | N | N | N | N | N | N | N |
| 7 | M | 27 | OD | Inherited | *FBN1* | c.5741G>A | p.C1914Y | S-T | Mild | N | Y | Y | N | N | N | N |
| 8 | M | 5 | OS | De novo | *FBN1* | c.1727G>A | p.C576Y | S-N | Moderate | N | N | N | N | N | N | N |
| 9 | M | 6 | OD | De novo | *FBN1* | c.3229T>C | p.S1077P | O | Moderate | N | N | Y | N | Y | N | N |
| 10 | F | 21 | OD | De novo | *FBN1* | c.5627G>A | p.C1876Y | I-T | Mild | N | Y | Y | N | N | N | N |
| 11 | F | 5 | OS | De novo | *FBN1* | c.4982G>A | p.G1661E | S-N | Mild | N | N | N | N | N | N | N |
| 12 | M | 7 | OD | Inherited | *FBN1* | c.6050G>A | p.C2017Y | S-N | Mild | N | N | Y | N | N | N | N |
| 13 | M | 22 | OD | De novo | *FBN1* | c.4096G>A | p.E1366K | S-N | Moderate | Y | N | Y | N | N | N | N |
| 14 | F | 10 | OS | De novo | *FBN1* | c.2432G>A | p.C811Y | S-N | Mild | N | N | N | N | N | N | N |
| 15 | M | 4 | OD | Inherited | *FBN1* | c.2741G>T | p.C914F | S-T | Mild | N | N | N | N | N | N | N |
| 16 | M | 30 | OS | De novo | *FBN1* | c.2534G>T | p.C845F | N | Mild | N | N | N | N | N | N | N |
| 17 | M | 33 | OS | Inherited | *FBN1* | c.1745G>A | p.C582Y | I-N | Moderate | N | N | N | N | N | N | N |
| 18 | F | 12 | OD | Inherited | *FBN1* | c.4285T>C | p.C1429R | S-N | Moderate | N | N | Y | N | N | N | N |
| 19 | F | 5 | OS | De novo | *FBN1* | c.3544T>G | p.C1182G | O | Moderate | N | N | N | N | N | N | N |
| 20 | M | 58 | OS | Inherited | *FBN1* | c.3148A>T | p.S1050C | S-N | Moderate | Y | N | Y | N | N | N | N |
| 21 | M | 11 | OD | Inherited | *FBN1* | c.3476G>T | p.C1159F | S | Severe | N | N | Y | N | Y | N | N |
| 22 | M | 5 | OS | De novo | *FBN1* | c.2848T>C | p.C950R | I-T | Severe | N | N | N | N | Y | N | N |
| 23 | F | 4 | OD | De novo | *FBN1* | c.2810G>A | p.C937Y | N | Moderate | N | Y | N | N | N | N | N |
| 24 | M | 6 | OS | De novo | *FBN1* | c.6697C>G | p.P2233A | S-N | Moderate | N | N | N | N | N | N | N |
| 25 | F | 8 | OS | Inherited | *FBN1* | c.2415T>G | p.C805W | N | Moderate | N | N | N | N | N | Y | N |
| 26 | M | 11 | OD | De novo | *FBN1* | c.4538G>C | p.C1513S | S-N | Moderate | N | N | N | N | N | N | N |
| 27 | M | 11 | OD | De novo | *FBN1* | c.1658T>G | p.F553C | I-N | Moderate | N | N | N | N | N | N | N |
| 28 | F | 9 | OD | De novo | *FBN1* | c.1709G>C | p.C570S | T | Severe | N | N | N | N | N | N | N |
| 29 | M | 7 | OS | Inherited | *FBN1* | c.4096G>A | p.E1366K | S-N | Mild | N | N | N | N | N | N | N |
| 30 | F | 4 | OD | De novo | *FBN1* | c.4454G>A | p.C1485Y | S-T | Mild | N | N | N | N | N | N | N |
| 31 | M | 6 | OS | De novo | *FBN1* | c.4537T>C | p.C1513R | S-N | Moderate | N | N | N | N | N | N | N |
| 32 | M | 4 | OD | Inherited | *FBN1* | c.1709G>C | p.C570S | O | Moderate | N | N | N | N | N | N | N |
| 33 | M | 26 | OS | Inherited | *FBN1* | c.1915T>C | p.C639R | S-N | Moderate | N | Y | N | N | N | N | N |
| 34 | M | 29 | OS | Inherited | *FBN1* | c.1600T>C | p.C534R | N | Moderate | N | N | Y | N | N | N | N |
| 35 | F | 6 | OS | Inherited | *FBN1* | c.1633C>T | p.R545C | S-N | Moderate | N | N | N | N | N | N | N |
| 36 | M | 10 | OS | De novo | *FBN1* | c.3545G>T | p.C1182F | O | Moderate | N | N | Y | N | N | Y | N |
| 37 | F | 8 | OS | De novo | *FBN1* | c.5801G>C | p.C1934S | N | Mild | N | N | N | N | N | N | N |
| 38 | F | 9 | OD | Inherited | *FBN1* | c.2810G>A | p.C937Y | I-N | Moderate | N | N | N | Y | N | N | N |
| 39 | F | 29 | OS | De novo | *FBN1* | c.6688T>C | p.C2230R | S | Mild | N | N | Y | N | N | Y | N |
| 40 | F | 7 | OD | De novo | *FBN1* | c.6379G>T | p.D2127Y | S-N | Moderate | N | Y | N | N | N | N | N |
| 41 | F | 4 | OS | De novo | *FBN1* | c.4096G>A | p.E1366K | S-N | Mild | N | N | N | N | N | N | N |
| 42 | F | 41 | OS | Inherited | *FBN1* | c.3725G>A | p.C1242Y | I-T | Moderate | N | N | N | N | N | N | N |
| 43 | F | 2 | OS | De novo | *FBN1* | c.2369G>A | p.C790Y | S-N | Mild | N | N | N | N | N | N | N |
| 44 | F | 4 | OD | De novo | *FBN1* | c.5800T>C | p.C1934R | S-N | Severe | N | N | Y | N | N | N | N |
| 45 | F | 4 | OD | Inherited | *FBN1* | c.1846G>A | p.E616K | O | Moderate | N | N | N | N | N | N | N |
| 46 | M | 7 | OS | Inherited | *FBN1* | c.1633C>T | p.R545C | N | Mild | N | N | N | N | N | N | N |
| 47 | F | 3 | OS | De novo | *FBN1* | c.3217G>A | p.E1073K | O | Severe | N | N | Y | N | Y | N | Y |
| 48 | F | 8 | OD | De novo | *FBN1* | c.3794G>A | p.C1265Y | I-N | Severe | N | N | N | N | N | N | N |
| 49 | F | 18 | OS | De novo | *FBN1* | c.5782T>C | p.C1928R | S | Mild | N | N | Y | N | N | N | Y |
| 50 | M | 5 | OS | Inherited | *FBN1* | c.4864T>C | p.C1622R | N | Severe | N | N | N | N | Y | N | N |
| 51 | F | 54 | OS | Inherited | *FBN1* | c.3244G>T | p.G1082C | I-N | Severe | Y | N | N | Y | Y | N | N |
| 52 | M | 3 | OS | De novo | *FBN1* | c.4120T>C | p.C1374R | S-N | Moderate | N | N | Y | N | N | N | N |
| 53 | M | 4 | OD | Inherited | *FBN1* | c.2804G>A | p.C935Y | S-N | Moderate | N | N | N | N | N | N | N |
| 54 | M | 4 | OS | De novo | *FBN1* | c.1948C>T | p.R650C | S-N | Moderate | N | N | N | N | N | N | N |
| 55 | M | 2 | OD | Inherited | *FBN1* | c.1670G>A | p.C557Y | N | Moderate | N | N | N | N | N | N | N |
| 56 | F | 4 | OS | Inherited | *FBN1* | c.1891A>C | p.T631P | S-N | Mild | N | Y | N | N | N | N | N |
| 57 | M | 4 | OS | Inherited | *FBN1* | c.1715A>C | p.D572A | S-N | Mild | N | N | N | N | N | N | N |
| 58 | M | 3 | OD | Inherited | *FBN1* | c.1879C>T | p.R627C | S | Moderate | N | N | N | N | N | N | N |
| 59 | F | 8 | OD | De novo | *FBN1* | c.1868G>T | p.C623F | S-T | Moderate | N | N | N | N | N | N | N |
| 60 | M | 6 | OD | De novo | *FBN1* | c.3921T>G | p.C1307W | S-N | Moderate | Y | N | N | N | N | N | N |
| 61 | M | 15 | OD | Inherited | *FBN1* | c.5503T>A | p.C1835S | I-N | Severe | N | N | Y | N | Y | Y | N |
| 62 | M | 31 | OS | De novo | *FBN1* | c.1774G>T | p.G592C | S-N | Moderate | Y | N | Y | N | N | N | N |
| 63 | M | 8 | OD | Inherited | *FBN1* | c.6158G>T | p.C2053F | S-N | Mild | N | N | Y | N | N | N | Y |
| 64 | M | 8 | OD | Inherited | *FBN1* | c.1948C>T | p.R650C | S-N | Moderate | N | N | N | N | N | N | N |
| 65 | M | 11 | OS | De novo | *FBN1* | c.2447G>C | p.C816S | S-N | Mild | N | N | N | N | N | N | N |
| 66 | F | 22 | OS | De novo | *FBN1* | c.2414G>A | p.C805Y | S | Mild | N | N | N | N | N | N | N |
| 67 | M | 6 | OS | Inherited | *FBN1* | c.4096G>A | p.E1366K | S-N | Mild | N | N | N | N | N | N | N |
| 68 | M | 6 | OD | De novo | *FBN1* | c.3997T>C | p.C1333R | S-T | Moderate | N | N | Y | N | N | N | Y |

^^^In the column of subluxation direction, N = nasal, T = temporal, S = superior, I = inferior and O = others. The others refer to lens tremor, anterior or posterior subluxation. M = male, F = female. OD = right eye, OS = left eye. MSP = microspherophakia. Y = yes, N = no.

**Supplementary Table S3.** Evaluation and previous publication of the reported variants.

| No. | Gene | Variant | | cbEGF-like no. | Conserved amino acid | Regions | AF_gnomAD | SIFT^#^ | PolyPhen^*^ | Condel^&^ | ACMG^^^ | Database^¶^ | References (PMID)^§^ |
| --- | --- | --- | --- | --- | --- | --- | --- | --- | --- | --- | --- | --- | --- |
|  |  | cDNA | Amino acid |  |  |  |  |  |  |  |  |  |  |
| 1 | *FBN1* | c.1904A>G | p.Y635C | 6 | F/Y | Ca^2+^-binding, C-terminal hairpin | 0 | D(0) | PosD(0.541) | D(0.628) | P | Recorded | 16222657 |
| 2 | *FBN1* | c.3244G>T | p.G1082C | 12 | - | Others | 0 | D(0) | ProD(0.993) | D(0.895) | P | Novel | - |
| 3 | *FBN1* | c.1633C>T | p.R545C | 4 | - | Others | 0 | D(0) | ProD(0.916) | D(0.811) | P | Recorded | 27353645, 15054843 |
| 4 | *FBN1* | c.5504G>A | p.C1835Y | 26 | C5 | Cysteine, C5 | 0 | D(0.01) | ProD(0.996) | D(0.867) | P | Recorded | 11700157, 10647894 |
| 5 | *FBN1* | c.3380G>T | p.G1127V | 13 | G1 | Others | 0 | D(0) | ProD(1) | D(0.945) | P | Recorded | - |
| 6 | *FBN1* | c.1638C>G | p.C546W | 4 | C3 | Cysteine, C3 | 0 | D(0) | ProD(0.997) | D(0.911) | P | Recorded | 17657824 |
| 7 | *FBN1* | c.5741G>A | p.C1914Y | 28 | C4 | Cysteine, C4 | 0 | D(0) | ProD(0.996) | D(0.906) | P | Novel | - |
| 8 | *FBN1* | c.1727G>A | p.C576Y | 5 | C1 | Cysteine, C1 | 0 | D(0) | ProD(0.982) | D(0.871) | P | Recorded | 18435798, 23684891 |
| 9 | *FBN1* | c.3229T>C | p.S1077P | 12 | - | Others | 0 | D(0) | ProD(0.989) | D(0.884) | LP | Recorded | - |
| 10 | *FBN1* | c.5627G>A | p.C1876Y | 27 | C5 | Cysteine, C5 | 0 | D(0) | ProD(0.996) | D(0.906) | P | Recorded | 16222657 |
| 11 | *FBN1* | c.4982G>A | p.G1661E | 24 | G1 | Others | 0 | D(0) | ProD(0.999) | D(0.935) | P | Novel | - |
| 12 | *FBN1* | c.6050G>A | p.C2017Y | 31 | C1 | Cysteine, C1 | 0 | D(0) | ProD(0.996) | D(0.906) | P | Novel | - |
| 13 | *FBN1* | c.4096G>A | p.E1366K | 19 | E | Ca^2+^-binding, N-terminal loop | 0 | D(0) | ProD(0.983) | D(0.873) | P | Recorded | 14695540, 24199744 |
| 14 | *FBN1* | c.2432G>A | p.C811Y | 9 | C1 | Cysteine, C1 | 0 | D(0) | ProD(0.994) | D(0.897) | P | Recorded | - |
| 15 | *FBN1* | c.2741G>T | p.C914F | 10 | C1 | Cysteine, C1 | 0 | D(0) | ProD(0.998) | D(0.919) | P | Novel | - |
| 16 | *FBN1* | c.2534G>T | p.C845F | 9 | C6 | Cysteine, C6 | 0 | D(0) | B(0.026) | N(0.446) | P | Recorded | 24199744 |
| 17 | *FBN1* | c.1745G>A | p.C582Y | 5 | C2 | Cysteine, C2 | 0 | D(0) | ProD(0.974) | D(0.861) | P | Novel | - |
| 18 | *FBN1* | c.4285T>C | p.C1429R | 20 | C4 | Cysteine, C4 | 0 | D(0) | ProD(0.993) | D(0.895) | P | Novel | - |
| 19 | *FBN1* | c.3544T>G | p.C1182G | 14 | C5 | Cysteine, C5 | 0 | D(0) | PosD(0.787) | D(0.739) | P | Novel | - |
| 20 | *FBN1* | c.3148A>T | p.S1050C | 11 | S | Ca^2+^-binding, C-terminal hairpin | 0 | D(0) | ProD(0.998) | D(0.919) | LP | Novel | - |
| 21 | *FBN1* | c.3476G>T | p.C1159F | 14 | C1 | Cysteine, C1 | 0 | D(0) | PosD(0.817) | D(0.754) | P | Novel | - |
| 22 | *FBN1* | c.2848T>C | p.C950R | 10 | C6 | Cysteine, C6 | 0 | D(0) | ProD(1) | D(0.945) | P | Recorded | - |
| 23 | *FBN1* | c.2810G>A | p.C937Y | 10 | C5 | Cysteine, C5 | 0 | D(0) | ProD(0.999) | D(0.935) | P | Novel | - |
| 24 | *FBN1* | c.6697C>G | p.P2233A | 34 | - | Others | 0 | D(0) | ProD(0.995) | D(0.902) | LP | Novel | - |
| 25 | *FBN1* | c.2415T>G | p.C805W | 8 | C6 | Cysteine, C6 | 0 | D(0) | ProD(0.997) | D(0.911) | P | Recorded | 20564469 |
| 26 | *FBN1* | c.4538G>C | p.C1513S | 22 | C5 | Cysteine, C5 | 0 | D(0) | B(0.18) | N(0.461) | P | Novel | - |
| 27 | *FBN1* | c.1658T>G | p.F553C | 4 | F/Y | Ca^2+^-binding, C-terminal hairpin | 0 | D(0) | ProD(0.997) | D(0.911) | P | Novel | - |
| 28 | *FBN1* | c.1709G>C | p.C570S | 4 | C6 | Cysteine, C6 | 0 | D(0) | ProD(0.977) | D(0.863) | P | Recorded | - |
| 29 | *FBN1* | c.4096G>A | p.E1366K | 19 | E | Ca^2+^-binding, N-terminal loop | 0 | D(0) | ProD(0.983) | D(0.873) | P | Recorded | 14695540, 24199744 |
| 30 | *FBN1* | c.4454G>A | p.C1485Y | 21 | C6 | Cysteine, C6 | 0 | D(0) | ProD(0.996) | D(0.906) | P | Recorded | - |
| 31 | *FBN1* | c.4537T>C | p.C1513R | 22 | C5 | Cysteine, C5 | 0 | D(0) | PosD(0.54) | D(0.627) | P | Recorded | 8136837 |
| 32 | *FBN1* | c.1709G>C | p.C570S | 4 | C6 | Cysteine, C6 | 0 | D(0) | ProD(0.977) | D(0.863) | P | Recorded | - |
| 33 | *FBN1* | c.1915T>C | p.C639R | 6 | C5 | Cysteine, C5 | 0 | D(0) | ProD(0.986) | D(0.879) | P | Novel | - |
| 34 | *FBN1* | c.1600T>C | p.C534R | 4 | C1 | Cysteine, C1 | 0 | D(0) | ProD(0.996) | D(0.906) | P | Recorded | 12938084 |
| 35 | *FBN1* | c.1633C>T | p.R545C | 4 | - | Others | 0 | D(0) | ProD(0.916) | D(0.811) | P | Recorded | 27353645, 15054843 |
| 36 | *FBN1* | c.3545G>T | p.C1182F | 14 | C5 | Cysteine, C5 | 0 | D(0) | PosD(0.894) | D(0.796) | P | Novel | - |
| 37 | *FBN1* | c.5801G>C | p.C1934S | 29 | C1 | Cysteine, C1 | 0 | D(0.03) | ProD(0.985) | D(0.796) | P | Recorded | - |
| 38 | *FBN1* | c.2810G>A | p.C937Y | 10 | C5 | Cysteine, C5 | 0 | D(0) | ProD(0.999) | D(0.935) | P | Novel | - |
| 39 | *FBN1* | c.6688T>C | p.C2230R | 34 | C4 | Cysteine, C4 | 0 | D(0) | ProD(0.996) | D(0.906) | P | Recorded | - |
| 40 | *FBN1* | c.6379G>T | p.D2127Y | 32 | D | Ca^2+^-binding, N-terminal loop | 0 | D(0) | ProD(0.999) | D(0.935) | P | Recorded | 11139245 |
| 41 | *FBN1* | c.4096G>A | p.E1366K | 19 | E | Ca^2+^-binding, N-terminal loop | 0 | D(0) | ProD(0.983) | D(0.873) | P | Recorded | 14695540, 24199744 |
| 42 | *FBN1* | c.3725G>A | p.C1242Y | 16 | C1 | Cysteine, C1 | 0 | D(0) | ProD(0.991) | D(0.889) | P | Recorded | 10486319 |
| 43 | *FBN1* | c.2369G>A | p.C790Y | 8 | C4 | Cysteine, C4 | 0 | D(0) | ProD(0.996) | D(0.906) | P | Recorded | - |
| 44 | *FBN1* | c.5800T>C | p.C1934R | 29 | C1 | Cysteine, C1 | 0 | D(0) | ProD(0.996) | D(0.906) | P | Recorded | - |
| 45 | *FBN1* | c.1846G>A | p.E616K | 6 | E | Ca^2+^-binding, N-terminal loop | 0 | D(0) | ProD(0.993) | D(0.895) | LP | Recorded | - |
| 46 | *FBN1* | c.1633C>T | p.R545C | 4 | - | Others | 0 | D(0) | ProD(0.916) | D(0.811) | P | Recorded | 27353645, 15054843 |
| 47 | *FBN1* | c.3217G>A | p.E1073K | 12 | E | Ca^2+^-binding, N-terminal loop | 0 | D(0) | PosD(0.824) | D(0.756) | P | Recorded | 7611299, 16596670 |
| 48 | *FBN1* | c.3794G>A | p.C1265Y | 16 | C5 | Cysteine, C5 | 0 | D(0) | ProD(0.991) | D(0.889) | P | Recorded | 18435798 |
| 49 | *FBN1* | c.5782T>C | p.C1928R | 28 | C6 | Cysteine, C6 | 0 | D(0) | ProD(0.996) | D(0.906) | P | Recorded | 7611299, 25101912 |
| 50 | *FBN1* | c.4864T>C | p.C1622R | 23 | C3 | Cysteine, C3 | 0 | D(0) | ProD(0.996) | D(0.906) | P | Recorded | 17657824 |
| 51 | *FBN1* | c.3244G>T | p.G1082C | 12 | - | Others | 0 | D(0) | ProD(0.993) | D(0.895) | P | Novel | - |
| 52 | *FBN1* | c.4120T>C | p.C1374R | 19 | C2 | Cysteine, C2 | 0 | D(0) | PosD(0.796) | D(0.744) | P | Novel | - |
| 53 | *FBN1* | c.2804G>A | p.C935Y | 10 | C4 | Cysteine, C4 | 0 | D(0.01) | ProD(0.999) | D(0.896) | P | Recorded | - |
| 54 | *FBN1* | c.1948C>T | p.R650C | 6 | - | Others | 0 | D(0) | ProD(0.99) | D(0.886) | P | Recorded | 28941062, 21932315 |
| 55 | *FBN1* | c.1670G>A | p.C557Y | 4 | C5 | Cysteine, C5 | 0 | D(0) | ProD(0.996) | D(0.906) | P | Recorded | - |
| 56 | *FBN1* | c.1891A>C | p.T631P | 6 | - | Ca^2+^-binding, C-terminal hairpin | 0 | D(0) | PosD(0.852) | D(0.772) | LP | Recorded | - |
| 57 | *FBN1* | c.1715A>C | p.D572A | 5 | D | Ca^2+^-binding, N-terminal loop | 0 | D(0) | ProD(0.979) | D(0.867) | LP | Novel | - |
| 58 | *FBN1* | c.1879C>T | p.R627C | 6 | - | Others | 0 | T(0.08) | PosD(0.446) | N(0.446) | P | Recorded | 17679947, 16220557 |
| 59 | *FBN1* | c.1868G>T | p.C623F | 6 | C2 | Cysteine, C2 | 0 | D(0) | ProD(0.986) | D(0.879) | P | Recorded | 18435798, 11251996 |
| 60 | *FBN1* | c.3921T>G | p.C1307W | 17 | C5 | Cysteine, C5 | 0 | D(0) | ProD(0.995) | D(0.902) | P | Recorded | - |
| 61 | *FBN1* | c.5503T>A | p.C1835S | 26 | C5 | Cysteine, C5 | 0 | D(0) | ProD(0.985) | D(0.877) | P | Novel | - |
| 62 | *FBN1* | c.1774G>T | p.G592C | 5 | G2 | Ca^2+^-binding, C-terminal hairpin | 0 | D(0) | ProD(1) | D(0.945) | P | Recorded | - |
| 63 | *FBN1* | c.6158G>T | p.C2053F | 31 | C6 | Cysteine, C6 | 0 | D(0) | B(0.1) | N(0.452) | P | Recorded | 10486319 |
| 64 | *FBN1* | c.1948C>T | p.R650C | 6 | - | Others | 0 | D(0) | ProD(0.99) | D(0.886) | P | Recorded | 28941062, 21932315 |
| 65 | *FBN1* | c.2447G>C | p.C816S | 9 | C2 | Cysteine, C2 | 0 | D(0) | ProD(0.978) | D(0.865) | P | Recorded | 14695540 |
| 66 | *FBN1* | c.2414G>A | p.C805Y | 8 | C6 | Cysteine, C6 | 0 | D(0) | ProD(0.996) | D(0.906) | P | Recorded | - |
| 67 | *FBN1* | c.4096G>A | p.E1366K | 19 | E | Ca^2+^-binding, N-terminal loop | 0 | D(0) | ProD(0.983) | D(0.873) | P | Recorded | 14695540, 24199744 |
| 68 | *FBN1* | c.3997T>C | p.C1333R | 18 | C2 | Cysteine, C2 | 0 | D(0) | PosD(0.721) | D(0.706) | P | Novel | - |

AF_gnomAD, allele frequency in gnomAD database.

^#^The variants were evaluated by SIFT and predicted as D, deleterious; T, tolerated. The scores were shown in the brackets.

^*^The variants were evaluated by PolyPhen and predicted as Pro, probably damaging; Pos, possibly damaging; B, benign. The scores were shown in the brackets.

^&^The variants were evaluated by Condel and predicted as D, deleterious; N, neutral. The scores were shown in the brackets.

^^^The variants were evaluated by the American College of Medical Genetics and Genomics (ACMG) guideline and classified as P, pathogenic; LP, likely pathogenic.

**Supplementary Table S4. Segregation data of the reported variants.**

| Proband No. | Family member No. | Relationship | Age (years old) | Phenotype | Gene | Variations | | State | Ghent2 | ACMG |
| --- | --- | --- | --- | --- | --- | --- | --- | --- | --- | --- |
|  |  |  |  |  |  | Nucleotide | Amino acid |  |  |  |
| 1 | 1-1 | Father | ≥20 | N | - | - | - | - | - | - |
|  | 1-2 | Mother | ≥20 | N | - | - | - | - | - | - |
| 2 | 2-1 | Son | <20 | ELS, G | *FBN1* | c.3244G>T | p.G1082C | Het | Causal | P |
|  | 2-2 | Husband | ≥20 | N | - | - | - | - | - | - |
| 3 | 3-1 | Father | ≥20 | N | - | - | - | - | - | - |
|  | 3-2 | Mother | ≥20 | N | - | - | - | - | - | - |
| 4 | 4-2 | Father | ≥20 | N | - | - | - | - | - | - |
|  | 4-3 | Mother | ≥20 | N | - | - | - | - | - | - |
| 5 | 5-1 | Father | ≥20 | N | - | - | - | - | - | - |
|  | 5-2 | Mother | ≥20 | N | - | - | - | - | - | - |
| 6 | 6-1 | Father | ≥20 | N | - | - | - | - | - | - |
|  | 6-2 | Mother | ≥20 | N | - | - | - | - | - | - |
| 7 | 7-1 | Mother | ≥20 | EL, RD | *FBN1* | c.5741G>A | p.C1914Y | Het | Causal | P |
|  | 7-2 | Father | ≥20 | N | - | - | - | - | - | - |
| 8 | 8-1 | Father | ≥20 | N | - | - | - | - | - | - |
|  | 8-2 | Mother | ≥20 | N | - | - | - | - | - | - |
| 9 | 9-1 | Father | ≥20 | N | - | - | - | - | - | - |
|  | 9-2 | Mother | ≥20 | N | - | - | - | - | - | - |
| 10 | 10-1 | Father | ≥20 | N | - | - | - | - | - | - |
|  | 10-2 | Mother | ≥20 | N | - | - | - | - | - | - |
| 11 | 11-1 | Father | ≥20 | N | - | - | - | - | - | - |
|  | 11-2 | Mother | ≥20 | N | - | - | - | - | - | - |
| 12 | 12-1 | Mother | ≥20 | EL | *FBN1* | c.6050G>A | p.C2017Y | Het | Causal | P |
|  | 12-2 | Father | ≥20 | N | - | - | - | - | - | - |
| 13 | 13-1 | Father | ≥20 | N | - | - | - | - | - | - |
|  | 13-2 | Mother | ≥20 | N | - | - | - | - | - | - |
| 14 | 14-1 | Father | ≥20 | N | - | - | - | - | - | - |
|  | 14-2 | Mother | ≥20 | N | - | - | - | - | - | - |
| 15 | 15-1 | Mother | ≥20 | ELS | *FBN1* | c.2741G>T | p.C914F | Het | Causal | P |
|  | 15-2 | Father | ≥20 | N | - | - | - | - | - | - |
| 16 | 16-1 | Father | ≥20 | N | - | - | - | - | - | - |
|  | 16-2 | Mother | ≥20 | N | - | - | - | - | - | - |
| 17 | 17-1 | Mother | ≥20 | MFS | *FBN1* | c.1745G>A | p.C582Y | Het | Causal | P |
| 18 | 18-1 | Mother | ≥20 | EL | *FBN1* | c.4285T>C | p.C1429R | Het | Causal | P |
|  | 18-2 | Father | ≥20 | N | - | - | - | - | - | - |
| 19 | 19-1 | Father | ≥20 | N | - | - | - | - | - | - |
|  | 19-2 | Mother | ≥20 | N | - | - | - | - | - | - |
| 20 | 20-1 | Daughter | ≥20 | EL | *FBN1* | c.3148A>T | p.S1050C | Het | Causal | LP |
| 21 | 21-1 | Father | ≥20 | MFS | *FBN1* | c.3476G>T | p.C1159F | Het | Causal | P |
|  | 21-2 | Mother | ≥20 | N | - | - | - | - | - | - |
|  | 21-3 | Brother | <20 | MFS | *FBN1* | c.3476G>T | p.C1159F | Het | Causal | P |
| 22 | 22-1 | Father | ≥20 | N | - | - | - | - | - | - |
|  | 22-2 | Mother | ≥20 | N | - | - | - | - | - | - |
| 23 | 23-1 | Father | ≥20 | N | - | - | - | - | - | - |
|  | 23-2 | Mother | ≥20 | N | - | - | - | - | - | - |
| 24 | 24-1 | Father | ≥20 | N | - | - | - | - | - | - |
|  | 24-2 | Mother | ≥20 | N | - | - | - | - | - | - |
| 25 | 25-1 | Father | ≥20 | EL | *FBN1* | c.2415T>G | p.C805W | Het | Causal | P |
|  | 25-2 | Mother | ≥20 | N | - | - | - | - | - | - |
| 26 | 26-2 | Father | ≥20 | N | - | - | - | - | - | - |
|  | 26-3 | Mother | ≥20 | N | - | - | - | - | - | - |
| 27 | 27-1 | Father | ≥20 | N | - | - | - | - | - | - |
|  | 27-2 | Mother | ≥20 | N | - | - | - | - | - | - |
| 28 | 28-1 | Father | ≥20 | N | - | - | - | - | - | - |
|  | 28-2 | Mother | ≥20 | N | - | - | - | - | - | - |
| 29 | 29-1 | Mother | ≥20 | EL | *FBN1* | c.4096G>A | p.E1366K | Het | Causal | P |
|  | 29-2 | Father | ≥20 | N | - | - | - | - | - | - |
| 30 | 30-1 | Father | ≥20 | N | - | - | - | - | - | - |
|  | 30-2 | Mother | ≥20 | N | - | - | - | - | - | - |
| 31 | 31-1 | Father | ≥20 | N | - | - | - | - | - | - |
|  | 31-2 | Mother | ≥20 | N | - | - | - | - | - | - |
| 32 | 32-1 | Father | ≥20 | MFS | *FBN1* | c.1709G>C | p.C570S | Het | Causal | P |
|  | 32-2 | Mother | ≥20 | N | - | - | - | - | - | - |
| 33 | 33-1 | Father | ≥20 | MFS | *FBN1* | c.1915T>C | p.C639R | Het | Causal | P |
|  | 33-2 | Mother | ≥20 | N | - | - | - | - | - | - |
|  | 33-3 | Sister | ≥20 | MFS | *FBN1* | c.1915T>C | p.C639R | Het | Causal | P |
| 34 | 34-1 | Son | <20 | MFS | *FBN1* | c.1600T>C | p.C534R | Het | Causal | P |
|  | 34-2 | Wife | ≥20 | N | - | - | - | - | - | - |
| 35 | 35-1 | Mother | ≥20 | ELS | *FBN1* | c.1633C>T | p.R545C | Het | Causal | P |
|  | 35-2 | Father | ≥20 | N | - | - | - | - | - | - |
| 36 | 36-1 | Father | ≥20 | N | - | - | - | - | - | - |
|  | 36-2 | Mother | ≥20 | N | - | - | - | - | - | - |
| 37 | 37-1 | Father | ≥20 | N | - | - | - | - | - | - |
|  | 37-2 | Mother | ≥20 | N | - | - | - | - | - | - |
| 38 | 38-1 | Father | ≥20 | N | - | - | - | - | - | - |
|  | 38-2 | Mother | ≥20 | EL | *FBN1* | c.2810G>A | p.C937Y | - | Causal | P |
|  | 38-3 | Brother | <20 | EL | *FBN1* | c.2810G>A | p.C937Y | - | Causal | P |
| 39 | 39-1 | Father | ≥20 | N | - | - | - | - | - | - |
|  | 39-2 | Mother | ≥20 | N | - | - | - | - | - | - |
| 40 | 40-1 | Father | ≥20 | N | - | - | - | - | - | - |
|  | 40-2 | Mother | ≥20 | N | - | - | - | - | - | - |
| 41 | 41-1 | Father | ≥20 | N | - | - | - | - | - | - |
|  | 41-2 | Mother | ≥20 | N | - | - | - | - | - | - |
| 42 | 42-1 | Father | ≥20 | MFS | *FBN1* | c.3725G>A | p.C1242Y | - | Causal | P |
|  | 42-2 | Mother | ≥20 | N | - | - | - | - | - | - |
|  | 42-3 | Son | <20 | MFS | *FBN1* | c.3725G>A | p.C1242Y | - | Causal | P |
| 43 | 43-1 | Father | ≥20 | N | - | - | - | - | - | - |
|  | 43-2 | Mother | ≥20 | N | - | - | - | - | - | - |
| 44 | 44-1 | Father | ≥20 | N | - | - | - | - | - | - |
|  | 44-2 | Mother | ≥20 | N | - | - | - | - | - | - |
| 45 | 45-2 | Father | ≥20 | EL, M | *FBN1* | c.1846G>A | p.E616K | - | Causal | LP |
|  | 45-3 | Mother | ≥20 | N | - | - | - | - | - | - |
| 46 | 46-1 | Father | ≥20 | NSCTD | *FBN1* | c.1633C>T | p.R545C | Het | Causal | P |
|  | 46-2 | Mother | ≥20 | N | - | - | - | - | - | - |
| 47 | 47-1 | Father | ≥20 | N | - | - | - | - | - | - |
|  | 47-2 | Mother | ≥20 | N | - | - | - | - | - | - |
| 48 | 48-1 | Father | ≥20 | N | - | - | - | - | - | - |
|  | 48-2 | Mother | ≥20 | N | - | - | - | - | - | - |
| 49 | 49-1 | Father | ≥20 | N | - | - | - | - | - | - |
|  | 49-2 | Mother | ≥20 | N | - | - | - | - | - | - |
| 50 | 50-1 | Mother | ≥20 | MFS | *FBN1* | c.4864T>C | p.C1622R | Het | Causal | P |
|  | 50-2 | Father | ≥20 | N | - | - | - | - | - | - |
|  | 50-3 | Maternal aunt | ≥20 | MFS | *FBN1* | c.4864T>C | p.C1622R | Het | Causal | P |
| 51 | 51-1 | Daughter | ≥20 | ELS, G | *FBN1* | c.3244G>T | p.G1082C | Het | Causal | P |
|  | 51-2 | Husband | ≥20 | N | - | - | - | - | - | - |
| 52 | 52-1 | Father | ≥20 | N | - | - | - | - | - | - |
|  | 52-2 | Mother | ≥20 | N | - | - | - | - | - | - |
| 53 | 53-1 | Mother | ≥20 | EL | *FBN1* | c.2804G>A | p.C935Y | Het | Causal | P |
|  | 53-2 | Father | ≥20 | N | - | - | - | - | - | - |
| 54 | 54-1 | Father | ≥20 | N | - | - | - | - | - | - |
|  | 54-2 | Mother | ≥20 | N | - | - | - | - | - | - |
| 55 | 55-1 | Father | ≥20 | MFS | *FBN1* | c.1670G>A | p.Cys557Tyr | Het | Causal | P |
|  | 55-2 | Mother | ≥20 | N | - | - | - | - | - | - |
| 56 | 56-1 | Mother | ≥20 | MFS | *FBN1* | c.1891A>C | p.T631P | Het | Causal | LP |
|  | 56-2 | Father | ≥20 | N | - | - | - | - | - | - |
| 57 | 57-1 | Father | ≥20 | M | *FBN1* | c.1715A>C | p.D572A | - | Causal | LP |
|  | 57-2 | Mother | ≥20 | N | - | - | - | - | - | - |
| 58 | 58-1 | Mother | ≥20 | ELS | *FBN1* | c.1879C>T | p.R627C | Het | Causal | P |
|  | 58-2 | Father | ≥20 | N | - | - | - | - | - | - |
| 59 | 59-1 | Father | ≥20 | N | - | - | - | - | - | - |
|  | 59-2 | Mother | ≥20 | N | - | - | - | - | - | - |
| 60 | 60-1 | Father | ≥20 | N | - | - | - | - | - | - |
|  | 60-2 | Mother | ≥20 | N | - | - | - | - | - | - |
| 61 | 61-1 | Mother | ≥20 | EL | *FBN1* | c.5503T>A | p.C1835S | Het | Causal | P |
|  | 61-2 | Father | ≥20 | N | - | - | - | - | - | - |
| 62 | 62-1 | Father | ≥20 | N | - | - | - | - | - | - |
|  | 62-2 | Mother | ≥20 | N | - | - | - | - | - | - |
| 63 | 63-1 | Father | ≥20 | EL | *FBN1* | c.6158G>T | p.C2053F | Het | Causal | P |
|  | 63-2 | Mother | ≥20 | N | - | - | - | - | - | - |
| 64 | 64-1 | Mother | ≥20 | MFS | *FBN1* | c.1948C>T | p.R650C | Het | Causal | P |
|  | 64-2 | Father | ≥20 | N | - | - | - | - | - | - |
| 65 | 65-1 | Father | ≥20 | N | - | - | - | - | - | - |
|  | 65-2 | Mother | ≥20 | N | - | - | - | - | - | - |
| 66 | 66-1 | Father | ≥20 | N | - | - | - | - | - | - |
|  | 66-2 | Mother | ≥20 | N | - | - | - | - | - | - |
| 67 | 67-1 | Father | ≥20 | EL | *FBN1* | c.4096G>A | p.E1366K | Het | Causal | P |
|  | 67-2 | Mother | ≥20 | N | - | - | - | - | - | - |
| 68 | 68-1 | Father | ≥20 | N | - | - | - | - | - | - |
|  | 68-2 | Mother | ≥20 | N | - | - | - | - | - | - |

ELS, ectopia lentis syndrome; MFS, Marfan syndrome; NSCTD, non-specific connective tissue disorder; N, normal; G, glaucoma; LP, likely pathogenic; P, pathogenic; EL, ectopia lentis; M, myopia over -3.0D; RD, postoperative retinal detachment; Het, heterozygote.

**Supplementary Table S5.** Ocular comorbidity and lens subluxation with *FBN1* mutations in cbEGF-like domains.

|  | | Functional regions (n = 57) | | Others  (n = 11) | *P* value |
| --- | --- | --- | --- | --- | --- |
|  |  | Cysteine  (n = 43) | Ca^2+^-binding  (n = 14) |  |  |
| Family history | Inherited | 17 | 6 | 5 | 0.913^*^ |
|  | *De novo* | 26 | 7 | 7 |  |
| Subluxation direction^^^ | Superior-nasal | 16 | 10 | 6 | 0.590 |
|  | Superior-temporal | 5 | 0 | 0 |  |
|  | Superior | 4 | 0 | 1 |  |
|  | Inferior-nasal | 4 | 1 | 1 |  |
|  | Inferior-temporal | 3 | 0 | 0 |  |
|  | Nasal | 7 | 0 | 2 |  |
|  | Temporal | 1 | 0 | 0 |  |
|  | Others | 3 | 2 | 2 |  |
| Subluxation severity | mild | 16 | 5 | 3 | 0.911 |
|  | moderate | 20 | 7 | 7 |  |
|  | severe | 7 | 1 | 2 |  |
| MSP | MSP | 4 | 1 | 3 | 0.267 |
|  | n.a. | 39 | 12 | 9 |  |
| Strabismus | Strabismus | 4 | 2 | 0 | 0.402 |
|  | n.a. | 39 | 11 | 12 |  |
| Staphyloma | Staphyloma | 15 | 4 | 1 | 0.221 |
|  | n.a. | 28 | 9 | 11 |  |
| Glaucoma | Glaucoma | 1 | 0 | 2 | 0.103 |
|  | n.a. | 42 | 13 | 10 |  |
| Ciliary body cyst | Ciliary body cyst | 4 | 0 | 0 | 0.448 |
|  | n.a. | 39 | 13 | 12 |  |
| Megalocornea | Megalocornea | 3 | 1 | 0 | 1.000 |
|  | n.a. | 40 | 13 | 11 |  |
| Total | | 43 | 13 | 12 | - |

^^^Others refer to lens tremor or anterior or posterior subluxation.

MSP = microspherophakia. n.a. = not applicable. The *P* values were reported by Chi-square test (marked with ^*^) or Fisher exact test. MSP = microspherophakia.

**Supplementary Table S6.** Patients with cbEGF-like mutations in functional regions other than Calcium-binding and cysteine regions had better visual outcomes.

|  | | Functional regions (n = 37) | | Others (n = 10) | *P* values^#^ |
| --- | --- | --- | --- | --- | --- |
|  |  | Cysteine (n = 28) | Ca^2+^-binding (n = 9) |  |  |
| Follow-up durations (months) | 1 (n = 6) | 3 | 2 | 1 | 0.936 |
|  | 3 (n = 3) | 3 | 0 | 1 |  |
|  | 6 (n = 9) | 5 | 2 | 1 |  |
|  | 12 (n = 9) | 6 | 2 | 1 |  |
|  | 24 (n = 20) | 11 | 3 | 6 |  |
| BCVA baseline  (LogMAR) | ≤0.3 (better, n = 9) | 4 | 2 | 3 | 0.516 |
|  | >0.3 (worse, n = 38) | 24 | 7 | 7 |  |
| BCVA at the last visit (LogMAR)^^^ | ≤0.3 (better, n = 41) | 23 | 8 | 10 | 0.418 |
|  | >0.3 (worse, n = 6) | 5 | 1 | 0 |  |

BCVA = best corrected visual acuity. ^^^This refers to the postoperative BCVA at the last visit. Visual records of patients with medical history of retinal detachment were excluded. LogMAR = 0.3 equals to 20/40, and LogMAR ≤0.3 indicates better visual capacity than LogMAR = 0.3.

^#^*P* values were reported by Fisher's exact test.
