# Supplementary material for: Cysteine Substitution and Calcium-Binding Mutations in FBN1 cbEGF-Like Domains Are Associated With Severe Ocular Involvement in Patients With Congenital Ectopia Lentis
Source: Front Cell Dev Biol. 2022 Feb 14;9:816397. doi: 10.3389/fcell.2021.816397 (PMC8882981; doi:10.3389/fcell.2021.816397)
Supplement: Supplementary file 2 [file DataSheet1.DOCX]

**Supplementary Figures and legends:**


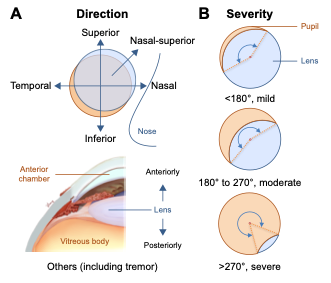


**Supplementary Figure S1.** Evaluation diagram of lens subluxation. The lens and the pupil are indicated by the blue and the orange circle, respectively. **(A)** Lens can subluxate into 8 directions (superior, inferior, temporal, nasal, nasal-superior, nasal-inferior, temporal-superior or temporal-inferior), axially (anteriorly or posteriorly) or tremor without obvious dislocation. This diagram depicted lens subluxation into the nasal-superior quadrant. **(B)** The severity of lens subluxation can be evaluated into 3 levels: mild, moderate and severe.

**Supplementary Figure S2.** Overview of selection criteria. MLPA = multiplex ligation-dependent probe amplification. NGS = next generation sequence, indel = insertion or deletion, Cys = cysteine, EGF = epidermal growth factor, cbEGF = calcium binding epidermal growth factor, LTBP = latent transforming growth factor β binding protein, TGFBP = transforming growth factor β binding protein.

**Supplementary Figure S3.** Correlations between ages and ocular biometrics. *P* values were reported by Spearman’s correlation test. AL = axial length, SA = spherical aberration, HOA = higher-order aberrations.

**Supplementary Figure S4.**

**(A)** Distribution of SA among different groups. SA was measured by a rotating Scheimpflug camera (Pentacam, Oculus GmbH, Wetzlar, Germany). **(B)** Distribution of ages among different groups. Differences among three mutations groups were analyzed with **Kruskal-Wallis test**. Post hoc comparisons were adjusted with **Bonferroni correction**. SA = spherical aberration.
